# Electroencephalographic Biomarkers in Tinnitus: A Narrative Review of Current Approaches and Clinical Perspectives

**DOI:** 10.3390/brainsci15121332

**Published:** 2025-12-14

**Authors:** Hyeonsu Oh, Dongwoo Lee, Jae-Kwon Song, Seunghyeon Baek, In-Ki Jin

**Affiliations:** 1 Department of Speech Pathology and Audiology, Graduate School, Hallym University, Chuncheon 24252, Republic of Korea; ohhs0130@gmail.com (H.O.); auditoryedu@hallym.ac.kr (D.L.); songjk1224@naver.com (J.-K.S.); qortmdgus501@naver.com (S.B.); 2 Division of Speech Pathology and Audiology, Research Institute of Audiology and Speech Pathology, College of Natural Sciences, Hallym University, Chuncheon 24252, Republic of Korea

**Keywords:** electroencephalography, tinnitus, biomarkers, neurophysiological markers, objective assessment

## Abstract

**Background/Objectives**: Tinnitus causes significant cognitive and emotional distress; however, its clinical assessment mostly relies on subjective measures without evaluation of objective indices. In this narrative review, we examined the potential of electroencephalography (EEG)-based neurophysiological markers as objective biomarkers in tinnitus assessment. **Methods**: The Web of Science, PubMed, EMBASE, and MEDLINE databases were searched to identify research articles on EEG-based analysis of individuals with tinnitus. Studies in which treatment and control groups were compared across four analytical domains (spectral power analysis, functional connectivity, microstate analysis, and entropy measures) were included. Qualitative synthesis was conducted to elucidate neurophysiological mechanisms, methodological characteristics, and clinical implications. **Results**: Analysis of 18 studies (n = 1188 participants) revealed that tinnitus is characterized by distributed neural dysfunction that extends beyond the auditory system. Spectral power analyses revealed sex-dependent, frequency-specific abnormalities across distributed brain regions. Connectivity analyses demonstrated elevated long-range coupling in high-frequency bands concurrent with diminished low-frequency synchronization. Microstate analyses revealed alterations in spatial configuration and transition probabilities. Entropy quantification indicated elevated complexity, particularly in the frontal and auditory cortices. **Conclusions**: EEG-derived neurophysiological markers demonstrate associations with tinnitus in group analyses and show potential for elucidating pathophysiological mechanisms. However, significant limitations, including low spatial resolution, small sample sizes, methodological heterogeneity, and lack of validation for individual-level diagnosis or treatment prediction, highlight the need for cautious interpretation. Standardized analytical protocols, larger validation studies, multimodal neuroimaging integration, and demonstration of clinical utility in prospective trials are required before EEG markers can be established as biomarkers for tinnitus diagnosis and management.

## 1. Introduction

Tinnitus is a condition characterized as the perception of sound without an external source. Tinnitus affects a significant proportion of adults worldwide, with a global prevalence estimated to be 14.4% (95% CI, 12.6–16.5%) [1,2]. Beyond auditory symptoms, tinnitus is commonly associated with cognitive impairments, such as memory and attention deficits, as well as emotional distress and sleep disturbances, all of which substantially diminish quality of life [3].

Clinical assessment of tinnitus is typically categorized into two domains: perception and reaction [4]. Perception encompasses the psychoacoustic properties of tinnitus, including pitch and loudness, measured through audiometric procedures. Reaction refers to the emotional and functional effects of tinnitus, often quantified using validated questionnaires such as the Tinnitus Handicap Inventory (THI) and Visual Analog Scales (VAS) [5,6]. Despite the widespread use and reliability of such subjective tools, universally accepted objective metrics for the evaluation of tinnitus are lacking, underscoring a gap in standardized outcome measures for tinnitus [4].

Subjective evaluation of tinnitus is strongly influenced by the psychological and contextual factors affecting the patient at the time of assessment, which lead to significant intra-individual variability and potential discrepancies between self-reported severity and actual therapeutic outcomes [7]. For instance, elevated depression scores are associated with inconsistent tinnitus severity ratings, a finding that illustrates the limitations of the current evaluation approaches [8]. The lack of objective biomarkers of tinnitus poses major challenges that hinder accurate diagnosis and evaluation of treatment efficacy in clinical research [9].

Electroencephalography (EEG) has emerged as a promising objective method for evaluating the underlying neurophysiological mechanisms of tinnitus. EEG offers noninvasive, temporally precise recordings of brain activity, enabling the application of advanced analytical approaches such as frequency band analysis, connectivity mapping, microstate segmentation, and entropy measurement [10,11,12]. These methodologies facilitate the exploration of underlying neural mechanisms and identification of potential biomarkers relevant to clinical practice [13].

Recent studies have revealed a variety of distinct EEG signatures in tinnitus populations, including increased gamma-band activity, reduced alpha suppression, altered functional connectivity in frontal and limbic networks, increased entropy in frontal regions, and atypical microstate dynamics [13,14,15]. The purpose of this review was to comprehensively analyze these diverse electrophysiological indicators identified in tinnitus research, with particular focus on an integrated analysis of neurophysiological markers with potential clinical relevance. By consolidating the findings obtained in previous studies using various methodologies, we aim to highlight consistent trends and explore future directions for objective tinnitus assessment in both research and clinical settings.

In this review, we distinguished between “neurophysiological markers” and “objective indicators” to clarify their clinical and research implications. “Neurophysiological markers” broadly refers to observable electrophysiological features (e.g., spectral power, entropy) that differ between tinnitus and control groups, whereas “objective indicators” are quantitative metrics that can be measured independently of patient self-reports. The term “biomarkers” was reserved for markers that have demonstrated sufficient sensitivity, specificity, and reliability to potentially aid diagnosis or prognosis in a clinical setting. Although we reviewed various markers and indicators, the primary goal of this synthesis was to assess their validity as true clinical biomarkers.

## 2. Materials and Methods

This narrative review provides an integrative synthesis of current evidence on EEG-based tinnitus research, emphasizing interpretive analysis of neurophysiological mechanisms, methodological frameworks, and clinical implications across four major EEG domains: spectral power, connectivity, microstates, and entropy. Relevant studies were identified from major international databases, including Web of Science, PubMed, EMBASE, and MEDLINE. Original articles on EEG analysis of patients with tinnitus published between 2000 and 2024 were considered eligible. Full-text qualitative analysis was conducted to identify conceptual and methodological patterns across the selected studies. Participants diagnosed with chronic subjective tinnitus (duration: >3 months) based on their audiological and clinical histories were classified as “tinnitus” groups [16]. Control groups were defined as participants who reported no tinnitus. Notably, matching hearing thresholds varied across studies.

Studies were considered eligible if they included human participants with tinnitus, were conducted using EEG-based analyses, and involved the analysis of at least one of the four abovementioned EEG domains. Review papers, case reports, animal or pediatric studies, non-English publications, and studies lacking EEG data or details of tinnitus-related outcomes were excluded.

## 3. Results

### 3.1. Study Selection and Review Structure

#### 3.1.1. Study Selection

A search of the Web of Science, PubMed, EMBASE, and MEDLINE databases identified original research articles on the evaluation of EEG-based measures in adults with chronic subjective tinnitus. Titles and abstracts were screened using the predefined eligibility criteria described in the Materials and Methods Section. Subsequently, the full texts of potentially eligible articles were assessed to confirm the presence of data on tinnitus-related EEG outcomes. Eighteen studies that met the eligibility criteria were included in the qualitative synthesis. These studies collectively included 1188 participants and covered four major EEG analytical domains: spectral power, functional connectivity, microstates, and entropy.

#### 3.1.2. Review Structure

This Results Section is organized into subsections focused on each of the four abovementioned EEG domains. For each domain, we first provide a concise overview of the relevant EEG methodology, followed by a focused synthesis of tinnitus-specific findings. The subsection on spectral power analyses (Section 3.2) provides a summary of frequency- and sex-dependent alterations in cortical oscillations. The subsection on connectivity studies (Section 3.3) addresses large-scale network reorganization and its relationship to tinnitus distress. The microstate analyses subsection (Section 3.4) describes changes in large-scale spatiotemporal network dynamics. The subsection on entropy-based studies (Section 3.5) addresses nonlinear complexity as a potential marker of tinnitus-related neural dysregulation. This domain-based structure was applied to clarify how different EEG metrics converge on a distributed network model of tinnitus.

### 3.2. EEG Power

#### 3.2.1. Definition and Theoretical Foundation

Scalp-recorded EEG signals have amplitudes of approximately 100 µV, and their frequencies range from 0.5 to 100 Hz depending on cortical activity [17]. The recorded EEG is a complex and chaotic signal composed of nonperiodic events (e.g., spikes, random noise), non-sinusoidal periodic rhythms (e.g., mu), and truly sinusoidal oscillations (e.g., alpha, beta), which makes direct analysis of the raw signal difficult [18]. However, as cortical activity patterns generally manifest as periodic sinusoidal signals, filtering, Fourier transformation, and wavelet analysis can isolate these sinusoidal components from the full EEG [19,20]. Subsequent EEG power spectral analysis quantitatively evaluates changes in each rhythm by dividing the isolated signal into delta (0.5–4 Hz), theta (4–7 Hz), alpha (8–12 Hz), beta (13–30 Hz), and gamma (30–80 Hz) bands, and computes the power distribution in each band [21].

#### 3.2.2. Interpretation Methodology

Interpretation of scalp-recorded EEG signals relies on several key methodological approaches used for extracting and quantifying frequency-specific activity. Filtering and time–frequency decomposition methods such as bandpass filtering, Fourier transformation, and wavelet analysis isolate and break down EEG signals into their constituent frequency components for power spectral quantification [19,20]. Spectral power analysis then quantifies neural activity by computing power spectral density (PSD), which can be expressed as absolute or relative power [22]. Collectively, these methodologies enable objective and quantitative assessment of cortical rhythms and functional brain states.

Filtering allows signals only within a specified frequency range to pass through [23]. For example, it can retain only the alpha band (8–12 Hz) while removing signals in other frequency ranges. Digital filtering operates in the time domain by convolving each data point and its neighbors with a set of weights representing the target signal pattern [24]. For instance, to detect a 10 Hz alpha rhythm, the filter weights are shaped as a 10 Hz sine wave; when the EEG data match that rhythm and phase, the convolution sum is maximized, whereas other frequencies yield smaller sums. By applying this convolution to each point in the time series, a continuous sequence of output values is generated, forming the filtered waveform.

Fourier transformation decomposes complex EEG signals into multiple sine waves, separating brain signals into various frequency components such as delta, theta, and alpha waves, thereby extracting the magnitude and phase of each frequency [25]. In addition, it involves the conversion of signal similarity along the time axis into energy distribution along the frequency axis by calculating the autocorrelation function to determine temporal similarity lag-by-lag. The values obtained are then multiplied by complex exponentials and integrated across the entire time lag range, thereby aggregating the contribution of each time lag at specific frequency bands and determining how concentrated the signal energy is [26]. Fourier transformation is well suited to stationary signal analysis, excels at isolating narrowband components such as pure sine waves, and offers superior computational efficiency for real-time applications [27]. However, it only reveals the average frequency components across the entire time interval but cannot determine “when” those frequencies occurred. For example, although Fourier transformation can identify 10 Hz alpha waves, it cannot distinguish whether they appeared 5 or 30 s after the onset of measurement. This makes it difficult to apply Fourier transformation in analyses of mental processes and brain responses to sensory stimuli that occur and disappear over short time periods. Additionally, Fourier transformation assumes stationarity, the premise that signals do not change over time. However, this assumption is violated in physiological signals such as EEG that continuously fluctuate over time, making proper analysis difficult [20]. The short-time Fourier transform (STFT) was developed to address these limitations. STFT segments the entire signal into short time windows and performs Fourier transformation on each segment to capture the time-varying spectrum [28]. STFT distinguishes segments through windowing centered on time, calculates the frequency-specific energy of each segment through Fourier transformation, and by repeating this process across different windows, can detect abnormal changes that occurred in brief moments [29].

Wavelet transformation is based on an analysis function with a zero mean to eliminate background signal components (capturing only pure signal fluctuations). Wavelet transformation operates within the limits of the uncertainty principle by transforming a single mother function in scale and time (automatically optimizing the time–frequency balance for each frequency), thereby achieving both spatial resolution at small scales and scale resolution at large scales, enabling the accurate decomposition of time–frequency characteristics and detection of singularities and abnormal changes through multiresolution signal analysis [30]. In wavelet analysis, the reference wavelet waveform is scaled to match the desired brain wave band, and the waveform is sequentially shifted along the time axis to measure the degree of overlap with the signal by integrating the multiplied values. Larger integration results indicate stronger manifestation of the corresponding frequency pattern at specific timepoints [31]. Wavelet analysis operates on multiple scales, allowing signals to be decomposed across various scales into signal sets ranging from low to high frequency bands [32]. Through the mother wavelet, which is the fundamental waveform of wavelet analysis, signals can be compared by adjusting size according to time, space, and scale, enabling simultaneous evaluation of how strong specific frequencies appear at specific times [33]. Wavelet analysis uses wide time windows for low frequencies and narrow time windows for high frequencies, allowing for visual identification of the frequency bands that are activated at specific timepoints following stimulus presentation. Due to this precision, wavelet analysis is useful for assessment of abnormal brain wave signals [20,27,34]. For example, when analyzing the enhancement of delta waves (1–4 Hz) at specific timepoints following stimulus presentation in EEG, wavelet transformation can track time-dependent frequency changes using large windows suited to low-frequency bands while simultaneously capturing instantaneous changes in high-frequency components using small windows, enabling precise identification of signal microstructure. In summary, filtering methods, Fourier transformation, and wavelet analysis all decompose EEG signals into sine wave components and are core methods used for comprehensive characterization of the time–frequency properties of the brain.

Extraction of sine wave components from raw EEG signals through filtering, Fourier transformation, or wavelet analysis generates a PSD that represents power per unit Hz. The absolute power of each band is derived by integrating all PSD values within the frequency range of that band, which represent the magnitude of cortical excitation or inhibition at each electrode [35,36,37]. Relative power is calculated by dividing the absolute power of each band by the sum of the absolute power values recorded across all analyzed bands, emphasizing the proportion of each rhythm relative to total energy, and enabling direct comparison of changes across bands [38].

Quantitative electroencephalography (QEEG) data can be interpreted according to the mechanism and intensity of power. For example, delta waves are prominent during early development and sleep states, indicating the integration of brain activity with maintenance of bodily homeostasis through associations between the autonomic nervous system and metabolic regulation. Elevated delta waves can be observed during hunger, sexual arousal, drug use, panic attacks, and chronic pain [39]. Frontal delta power modulates sleep and depressive symptoms and reflects psychological distress and sleep states in proportion to its amplitude [40,41]. Strong delta slowing below 1.4 Hz in the temporal lobe is linked to hippocampal interictal epileptiform discharges, which are abnormal electrical activity between seizures, making it a marker of temporal lobe epilepsy [42].

Theta oscillations are associated with verbal memory recall, attention, and executive function, reflecting cognitive processing [43]. Sustained elevation of theta power in alcoholics suggests impaired central nerve system information processing capacity and imbalanced cortical excitation and inhibition [44]. In Alzheimer’s dementia (AD), temporal high-frequency power decreases while theta power increases globally, indicating that elevation of relative theta power may serve as an early marker of AD [45].

Alpha rhythms are related to attention, emotion, and creative thinking, and regulate cortical excitability via inhibition. Upper alpha rhythms suppress irrelevant inputs, whereas lower alpha rhythms permit greater excitation [46,47,48]. Increased alpha power weakens the connectivity between the primary visual cortex and occipital regions, reflecting functional inhibition of the visual network [47]. Adults with autism spectrum disorder show reduced alpha power in eyes-open conditions, which correspond to diminished inhibitory control and heightened perceptual focus [49]. Alpha power decreases during creative idea generation to allow for a broader range of thought and novel associations [48]. Thus, alpha oscillations help suppress non-task-relevant areas while enhancing processing in task-relevant regions [50].

Beta activity is linked to rest and sleep, reflecting both cortical arousal and sleep-protective processes [51]. Alcoholics exhibit elevated resting-state beta power, which indicates a disruption of cortical excitation–inhibition homeostasis [52]. Patients with insomnia show increased beta power at sleep onset and during non-rapid eye movement (NREM) sleep compared to healthy sleepers or depressed insomniacs. Notably, higher beta power predicts worse sleep quality [53].

Gamma oscillations underlie cognitive task performance, appearing during attention- and memory-related processes such as learning and reading [54]. Gamma activity is markedly altered in psychiatric and neurological disorders such as schizophrenia and AD. Patients with schizophrenia show reduced gamma amplitude while experiencing symptoms and heightened gamma amplitude during hallucinations. Patients with AD exhibit overall reduction in gamma amplitude. Patients with epilepsy display increased gamma amplitude, possibly reflecting both cortical hyperexcitability and perceptual distortions such as déjà vu. In addition, patients with attention-deficit/hyperactivity disorder exhibit elevated gamma amplitude [55]. These frequency-specific power changes reflect distinct neural mechanisms and may serve as neurophysiological biomarkers.

#### 3.2.3. Tinnitus-Specific Research Evidence

This section describes the synthesis of findings from six studies (N = 568 participants) on EEG power alterations in tinnitus populations (Table 1).

In QEEG analysis, tinnitus is detected as abnormal EEG signal patterns across diverse mechanisms beyond the auditory pathway. This suggests that tinnitus is not merely a localized issue of the auditory cortex but rather a problem that affects multiple brain regions and requires examination of the overall changes in EEG patterns [56]. Individuals with tinnitus tend to show abnormal QEEG power indices recorded at multiple electrode sites, unequal power distribution across multiple frequency bands, and neurophysiological patterns that differ from those of control groups [57]. In addition, they tend to exhibit power changes in delta, theta, alpha, and beta frequency bands across several brain regions including the temporal lobe, insular cortex, and limbic system, with potentially greater changes in eyes-open EEG indices [56,57,58,59]. EEG distribution spans multiple cortical regions, with abnormal activation across the delta, theta, alpha, and beta frequencies observed in the frontal, temporal, parietal, central, and occipital lobes [57]. Notably, abnormal activation in the insula is consistently reported in both eyes-open and eyes-closed conditions [56].

Sex-dependent patterns have been observed in the EEG characteristics of tinnitus populations. Females tend to show consistent power elevation across the theta, alpha, and beta frequency bands. However, some reports have indicated that males with tinnitus exhibit a power reduction in delta, theta, alpha, and beta bands, whereas others have demonstrated that males show elevated delta, theta, and beta power. These conflicting findings suggest that males with tinnitus may exhibit elevated or reduced EEG power relative to normal controls [56,58,59]. These discrepancies may be attributable to moderating variables that were not uniformly controlled across studies. Specifically, tinnitus laterality (unilateral vs. bilateral) has been shown to differentially modulate resting-state power. In addition, the degree of hearing loss, often more severe in male cohorts, may act as a confounding factor that drives cortical reorganization [60]. Furthermore, methodological differences, such as the specific cut-off frequencies used to define delta and theta bands and the choice of reference electrode, likely contributed to the divergent results observed in male participants. It is crucial to acknowledge these contradictions in reported findings, particularly regarding delta and theta power changes. Although some studies have demonstrated increased low-frequency power indicative of deafferentation, others have shown reductions or no change. These inconsistencies likely stem from differences in tinnitus chronicity (acute vs. chronic plastic changes). For instance, Vanneste et al. [61] demonstrated that acute and chronic tinnitus exhibit distinct spectral signatures, suggesting that the underlying neural drivers shift over time from auditory cortex hyperactivity to distributed network dysrhythmia.

Individuals with bilateral, right-sided, or left-sided tinnitus may exhibit distinct EEG power distributions, with males demonstrating consistent reduction in delta, theta, and alpha power across all laterality presentations, and females showing elevated alpha power regardless of laterality, heterogeneity in beta power, elevation of right-sided tinnitus, and reduction in left-sided tinnitus [58].

EEG responses categorized according to the presence and level of tinnitus distress differ across frequency bands and brain regions. In a previous study on EEG-based analysis of tinnitus presence and distress, individuals with high tinnitus distress exhibited elevated beta power with a strong positive correlation in the frontal lobe, whereas individuals with high tinnitus presence demonstrated elevated delta, alpha, and gamma power with a weak positive correlation in the temporal auditory cortex [62]. Although individuals with tinnitus do not show alterations in gamma power relative to normal controls, the observation of gamma power changes in individuals with high tinnitus presence suggests that elevated tinnitus presence may exceptionally influence gamma activity [56]. Moreover, EEG patterns and distribution may vary according to the degree of tinnitus distress and presence. In tinnitus treatment and rehabilitation, monitoring changes in specific EEG patterns and distribution may demonstrate utility as an objective approach to evaluating and tracking changes in tinnitus distress [63].

In tinnitus research, indicators of tinnitus relief are commonly assessed using subjective questionnaires such as the THI, Tinnitus Questionnaire (TQ), and VAS [64]. Repetitive transcranial magnetic stimulation (rTMS) targeting the auditory and prefrontal cortices has been shown to produce moderate tinnitus relief, as reflected in improvements on these scales [65]. Supporting the neurophysiological basis of these clinical effects, EEG studies have demonstrated that rTMS can modulate cortical oscillations depending on the stimulation site. Left temporal stimulation increases delta and theta power while reducing beta power in the frontal lobe, whereas right prefrontal stimulation tends to reduce beta and gamma power in the right temporal lobe [66]. These findings suggest that tinnitus relief may be accompanied by elevated delta and theta activity, together with reduced beta and gamma activity in specific cortical regions. Such EEG power changes could serve as potential objective biomarkers of treatment responsiveness and therapeutic outcomes.

**Table 1:** Summary of studies on EEG power analysis in tinnitus populations (the reviewed studies included those that reported quantitative power spectral analyses of patient and control groups, published 2000–2024).

Author(s) & Year of Publication	EEG Recording Configuration	Experiment Conditions	EEG Analysis Method	Study Population (Sample Size)	Frequency Band(s) /Target Regions	Main Findings	Key EEG Indicators Identified in Tinnitus Studies
Moazami-Goudarzi et al., 2010 [56]	60 electrodes (extended 10–20 system)	EC, EO	Filtering (0.3–100 Hz), Fourier transformation, low resolution electromagnetic tomography analysis	TI(M): 8 NC: 15	δ (2–4 Hz), θ (4–8 Hz), α (8–12 Hz), lower-β (12–18 Hz), upper-β (18–30 Hz), γ (30–100 Hz)/BA 6, 9, 21, 22, 23, 24, 29, 30, 32, 41, 42, insula, parahippocampal gyrus, prefrontal, right fronto-central regions	TI(M): Average ↑δ, θ, β power *; EC ↑δ power in BA 21, 22, 41, 42, insula *; EC ↑θ power in BA 21, 22, 23, 29, 30, 41, 42, insula *; EC ↑β power in BA 6, 9, 24, 32, insula *; EO ↑δ power in BA 21, 22, 23, 29, 30, 41, 42, insula, parahippocampal gyrus *; EO ↑θ power in BA 21, 22, 23, 29, 30, 41, 42, insula, parahippocampal gyrus *; EO ↑α power in BA 21, 22, 41, 42, insula, prefrontal *; EO ↑β power in right fronto-central regions *	TI(M) characteristics: Global EEG pattern (TI: Average ↑δ, θ, β power); EC EEG neurobiological regions (TI: ↑δ, θ, β in BA 6, 9, 21, 22, 23, 24, 29, 30, 32, 41, 42, insula); EO EEG neurobiological regions (TI: ↑δ, θ, α, β in BA 21, 22, 23, 29, 30, 41, 42, insula, parahippocampal gyrus, prefrontal, right fronto-central)
Shulman et al., 2006 [57]	19 electrodes (10–20 system)	EC, EO	Filtering (0.5–32 Hz), QEEG	TI(M): 42 TI(F): 19	δ (1.5–3.5 Hz), θ (3.5–7.5 Hz), α (7.5–12.5 Hz), β (12.5–25 Hz), γ (25–50 Hz)/frontal, temporal, parietal, central, occipital	TI(F): ↑θ, ↑α power * TI: abnormal QEEG δ, θ, α, β power in frontal, temporal, parietal, central, occipital regions	TI characteristics: Sex-dependent pattern (F: ↑θ, ↑α power); EEG Neurobiological regions (TI: Abnormal QEEG δ, θ, α, β power across frontal, temporal, parietal, central, occipital regions demonstrating distributed cortical dysrhythmia)
Weiler et al., 2000 [58]	19 electrodes (10–20 system)	EC, RS	Filtering (2–32 Hz), QEEG	TI(M)-B/R/L: 19/29/45 TI(F)-B/R/L: 15/13/30 NC(M): 35 NC(F): 20	δ (2–4 Hz), θ (4–7 Hz), α (8–13 Hz), β (14–21 Hz)/frontocentral, central, parietal, temporal, occipital	TI(M): ↓δ, power in B ***, R ***, L ***; ↓θ power in B **, R **, L **; ↓α, ↓β power in B ***, L *** TI(F): ↑δ, ↑θ power L ***; ↑α power in B **, R **, L ***; ↑β power in R ***; ↓β power in L ***	TI characteristics: Sex-dependent pattern (M: ↓δ, θ, α, β; F: ↑δ, θ, α with β asymmetry); Laterality-specific EEG signatures (TI: B ≠ R ≠ L)
Weiler & Brill, 2004 [59]	19 electrodes (10–20 system)	EC	Filtering (2–32 Hz), QEEG	TI(M): 195 TI(F): 109 NC(M): 94 NC(F): 61	δ (0–4 Hz), θ (4–7 Hz), α (8–13 Hz), β (14–21 Hz)/frontopolar, frontal, central, parietal, temporal, occipital regions	TI(M): Average ↓δ, θ, α power ***; Average ↓β power * TI(F): Average ↑θ power *; Average ↑α power ***; Average ↑β power **	TI characteristics: Sex-dependent pattern (M: ↓δ, θ, α, β; F: ↑θ, α, β)
Meyer et al., 2014 [62]	129 electrodes (Dense array)	EC, EO	Filtering (0.5–100 Hz), Fourier transformation	TI: 24 NC: 24	δ (0.5–4 Hz), α (9–13 Hz), upper-β (20–25 Hz), lower-γ (30–40 Hz)/frontal, temporal auditory cortex	EC high distress TI ↑upper-β power↔strong positive correlation in frontal lobe EC high presence TI ↑δ, α, lower-γ power↔weak positive correlation in temporal auditory cortex	TI handicap: Tinnitus distress (TI: ↑upper-β power, strong positive correlation in frontal lobe); Tinnitus presence (TI: ↑δ, α, lower-γ power, weak positive correlation in temporal auditory cortex)
Schecklmann et al., 2015 [66]	63 electrodes (10–20 system)	EC, rTMS	Filtering (1–45 Hz), fast Fourier transformation	TI: 20 Sham-NC: 20	δ (2–3.5 Hz), θ (4–7.5 Hz), lower-α (8–10 Hz), upper-α (10.5–12.5 Hz), lower-β (13–18 Hz), mid-β (18.5–21 Hz), upper-β (21.5–30 Hz)/frontal, temporal	Left temporal stimulation: ↑δ, θ, ↓mid-β power in frontal lobe ** Right prefrontal stimulation: ↓high-β, γ power in right temporal lobe **	TI relief: left temporal stimulation (TI: ↑δ, θ, ↓mid-β power in frontal lobe); right prefrontal stimulation (↓upper-β, γ power in right temporal lobe)

Methodological considerations: Sample sizes, electrode densities, and statistical rigor vary across studies. Effect sizes were not consistently reported in the original publications, limiting quantitative synthesis. Readers should interpret findings in consideration of these methodological variations. B, bilateral tinnitus; EC, eyes closed; EO, eyes open; F, female; L, left tinnitus; M, male; NC, normal control group; QEEG, quantitative electroencephalographic; R, right tinnitus; RS, resting state; rTMS, repetitive transcranial magnetic stimulation; TI, individuals with tinnitus; α, alpha band; β, beta band; γ, gamma band; δ, delta band; θ, theta band; ↑/↓ denotes a significant difference (higher/lower) between compared groups; ↔ denotes bidirectional connectivity. *: *p* < 0.05; **: *p* < 0.01; ***: *p* < 0.001.

### 3.3. EEG Connectivity

#### 3.3.1. Definition and Theoretical Foundation

Brain connectivity refers to the quantitative description of interactions among spatially distinct brain regions, including the anatomical pathways linking them, the statistical and temporal dependencies of neurophysiological signals, and the causal directional influences underlying their communication [67]. Brain connectivity is commonly categorized into three types: structural (or neuroanatomical), functional, and effective connectivity [68].

Structural connectivity refers to the anatomically defined physical connections between brain regions [69], including synaptic linkages and neural fiber tracts. The direct monosynaptic projections that connect the hippocampus and prefrontal cortex is an example of structural connectivity [70]. Functional connectivity is defined as the non-directional correlation among anatomically distinct brain regions, reflecting statistically significant coupling that represents direct or indirect interregional communication during the resting state [71,72]. Increased functional connectivity between the hippocampus and parahippocampal gyrus during the recall phase represents non-directional coupling between memory-related regions [73]. Effective connectivity is a model parameter that explains how one neural system influences another, accounting for interdependencies among brain regions and representing the directional influence from one region to another [74,75]. At a certain stage after stroke, effective connectivity is enhanced as the motor cortex in the unaffected hemisphere supports the damaged side, reflecting a compensatory neural mechanism by which the brain attempts to restore impaired motor function [76].

#### 3.3.2. Interpretation Methodology

Structural connectivity in brain measurements refers to the physical architecture of the white matter tracts linking distinct cortical and subcortical regions. It is typically derived from diffusion-based magnetic resonance imaging techniques, such as diffusion tensor imaging or diffusion spectrum imaging, which visualize the macrostructural organization of the brain’s white matter pathways. EEG does not directly capture these anatomical connections; rather, it infers the temporal coordination of neuronal populations through scalp-recorded electrophysiological signals. Nevertheless, EEG allows for indirect examination of large-scale functional interactions by capturing synchronized electrical activity at millisecond temporal resolutions, typically assessed through condition-wise contrasts and statistical inference [77]. EEG-based studies primarily focus on functional connectivity, which quantifies statistical dependencies among distributed neural events, and effective connectivity, which models the directional causal interactions between neural systems [75].

Functional connectivity is widely used to assess the degree of synchronization and integration across distributed brain networks [75]. It is commonly analyzed using various computational techniques, including the phase-locking value (PLV), partial correlation coefficient, and coherence measures. PLV represents the consistency of phase alignment between two signals at a specific frequency. A value close to 1 denotes highly stable phase relationships across trials, indicating strong synchrony, whereas a value close to 0 reflects random phase variability. As PLV captures phase consistency independent of amplitude fluctuations, it has been extensively used to characterize large-scale neural synchronization [77,78]. However, given that PLV reflects only phase information, it is difficult to determine whether the synchronization it indicates arises solely from phase alignment or also involves amplitude-based dynamics [79].

To complement this limitation, correlation-based approaches such as partial correlation coefficient have been employed to capture statistical dependencies beyond pure phase relationships. The partial correlation coefficient quantifies the statistical dependence between two brain regions while controlling for the influence of all other regions, thereby providing a more direct estimate of connectivity strength [80]. Partial correlation coefficients range from −1 to +1, with positive values indicating co-varying activity, negative values representing inverse relationships, and zero denoting conditional independence from all other regions [81]. Notably, negative partial correlations should not be assumed to reflect inhibitory coupling; rather, they should be interpreted with caution by adjusting the conditioning set, controlling for potential confounders, and seeking converging evidence from complementary measures [82].

These functional connectivity metrics can be used in a complementary manner, and among them, coherence stands out as a spectrum-based approach that integrates both phase and amplitude information. Coherence quantifies frequency-specific associations between regions, with values ranging from 0 to 1. A value close to 1 indicates a near-perfect linear relationship. Given that coherence reflects consistency in both phase and amplitude, it is widely used to assess the strength of functional coupling between neural signals [83]. Increased coherence within a given frequency band suggests stable coupling, whereas low values imply weak or absent interactions [84]. Notably, as coherence combines phase and amplitude, distinguishing their separate contributions can be challenging [78]. Therefore, valid interpretation requires recognition of these mixing effects and, when necessary, using coherence in conjunction with other connectivity measures or complementary analyses.

Traditional functional connectivity metrics such as PLV, partial correlation coefficient, and coherence remain widely used because they provide established frameworks for quantifying large-scale neural synchrony and enable straightforward comparison of findings. However, these measures may incorporate zero-lag correlations arising from volume conduction. This means that coupling estimates can partially reflect shared signal origins rather than true neural communication. Delayed-interaction approaches, including weighted phase lag index, imaginary coherence, and lagged coherence, have been adopted to address this issue because they suppress zero-lag components and better capture physiologically meaningful interactions [85,86]. Nonetheless, as these indices remain inherently non-directional, effective connectivity methodologies are still required for inference of causal influences in neural systems.

Functional connectivity describes undirected statistical dependencies, whereas effective connectivity specifies directional causal influences among neural systems. Effective connectivity is a model-based inference that depicts how one neural system influences another, representing directional influence while taking interdependencies among brain regions into account [75]. These approaches are broadly categorized into data-driven and model-based methods [87]. A representative data-driven approach, Granger causality, estimates directional influences based purely on improvements in time-series prediction; however, its estimates can be distorted by non-stationarity and simultaneous covariation. In contrast, model-based approaches such as Dynamic Causal Modeling infer effective connectivity by integrating existing knowledge about network structure into the model; however, they depend on model assumptions and parameter identifiability [87].

Given that connectivity values are highly dependent on preprocessing choices and model assumptions, they are typically interpreted in terms of relative contrasts or directional trends rather than as absolute physiological constants [77]. In empirical EEG studies, connectivity metrics interpreted through these approaches are used to identify frequency-specific or condition-dependent variations across brain networks [88].

EEG-based connectivity analysis has been extensively utilized to detect frequency-specific large-scale network abnormalities across a variety of neurological and psychiatric disorders. In patients with AD and mild cognitive impairment, functional connectivity within the alpha frequency band is markedly reduced relative to that in healthy controls [89,90], reflecting a breakdown in the synchronous activation of large-scale brain networks and supporting the characterization of AD as a connectivity disorder. In contrast, schizophrenia is characterized by particularly aberrant activity within the gamma frequency range. Some studies have indicated widespread increase in gamma-band connectivity across cortical regions [91], whereas others have demonstrated reductions confined to specific areas such as the right temporoparietal cortex [92]. This variability has been attributed to methodological differences, dysfunction within inhibitory networks that modulate gamma oscillations, and neuro-physiological diversity across patient populations [93]. Notably, abnormalities in gamma rhythms are believed to reflect impaired inhibitory control resulting from diminished N-methyl-D-aspartate receptor function, a central mechanism underlying the excitatory–inhibitory imbalance and a core pathophysiological feature of schizophrenia [94]. Taken together, these frequency-specific connectivity alterations suggest abnormal coordination of large-scale brain networks, reflecting both disorder-specific pathophysiology and broader disruptions in neural communication dynamics.

#### 3.3.3. Tinnitus-Specific Research Evidence

This section describes the synthesis of findings from five studies (N = 482 participants) on EEG connectivity alterations in tinnitus populations (Table 2).

Tinnitus is characterized by aberrant EEG connectivity patterns that extend beyond the auditory pathway, which suggest a distributed network dysfunction rather than a localized abnormality in the auditory cortex [95,96]. Frequency-specific analyses have shown that individuals with tinnitus exhibit increased connectivity in beta and gamma bands, and reduced connectivity in low-frequency bands such as delta and alpha [95]. In addition, large-scale brain network analyses have indicated that long-range lagged phase synchronization is enhanced across multiple frequency bands. Network activity tends to become more structured and regular in low-frequency oscillations, and more disorganized and random in high-frequency ranges. These patterns indicate impaired regulation of functional integration and segregation within the brain. Therefore, alterations in EEG connectivity patterns may have some potential as objective neurophysiological markers of tinnitus severity and treatment responsiveness [97].

Beyond these global connectivity alterations, EEG-based connectivity patterns are also modulated by demographic factors such as sex, which may contribute to sex-specific emotional responses to tinnitus [98]. Reports have indicated that female patients show stronger connectivity in the alpha frequency band than male patients, particularly across circuits involving the parahippocampus, subgenual anterior cingulate cortex, left insula, orbitofrontal cortex, and secondary auditory cortex. This may suggest enhanced coupling between auditory processing and emotional regulation regions in females. However, it is important to note that these findings were obtained after multiple statistical comparisons across extensive network nodes. Although these sex-specific effects are notable, the statistical powers of these studies in relation to sex-by-network interactions may be limited by their sample sizes. Future studies that involve rigorous corrections for multiple comparisons are necessary to validate this reported female-specific alpha hyperconnectivity. Such sex-specific EEG connectivity patterns may serve as neurophysiological biomarkers that could facilitate our understanding of differential tinnitus-related emotional responses between males and females.

The severity of tinnitus-related distress seems to be accompanied by distinct changes in brain connectivity. Patients with moderate to severe tinnitus exhibit increased beta band coupling between the parahippocampal gyrus and the posterior cingulate cortex (PCC), and enhanced functional connectivity in the gamma band between the auditory cortex and the insula [96]. In addition, this insula-centered network exhibits a significant positive correlation with THI scores [96]. This finding suggests that the insula may serve as a key hub that mediates interactions between auditory input and networks involved in emotion and attention. Furthermore, distress-dependent alterations in default mode network (DMN) connectivity have been reported. Patients with higher distress levels show reduced alpha activity in the precuneus and the PCC, indicating impaired intrinsic network regulation [98].

From the perspective of evaluating therapeutic effects in tinnitus, normalization of brain network connectivity appears to be associated with the alleviation of tinnitus-related distress [97,99]. Real-time source-localized neurofeedback of the PCC achieves significant reductions in tinnitus-related distress, accompanied by normalization of DMN connectivity patterns, despite producing no significant change in neural activity within the target region itself [97]. These reductions in distress are accompanied by decreased alpha-band functional connectivity between the PCC and dorsal/subgenual anterior cingulate cortices, as well as between the subgenual anterior cingulate and parahippocampal regions, along with significant reductions in alpha–beta and alpha–gamma cross-frequency coupling, which normalizes to levels observed in healthy controls. Furthermore, abnormalities in alpha- and beta-band functional connectivity among regions such as the anterior cingulate cortex, insula, orbitofrontal cortex, and parahippocampal areas have been linked to the severity of tinnitus distress. This indicates that altered network interactions play a crucial role in tinnitus distress [99]. Therefore, these patterns of frequency-specific and connectivity-normalized brain dynamics may have potential as objective neurophysiological biomarkers of treatment-related improvements in distressed patients with tinnitus [97,99].

### 3.4. EEG Microstates

#### 3.4.1. Definition and Theoretical Foundation

Microstates are conceptualized as transient spatial configurations of scalp potential distributions observed in EEG signals, representing segmentable components of thought processes (building blocks) and atoms of thought [100]. Global field power (GFP) was computed in early studies and defined as the standard deviation of scalp potential maps at individual timepoints [101]. These studies also identified microstate intervals centered around timepoints of local GFP maxima. Each interval is characterized by the locations of maximal and minimal potential extrema, reflecting a spatially stable scalp topography that persistently holds until a discontinuous, jump-like transition to a new spatial configuration occurs [102]. Microstates exhibit polarity invariance as spatial topographical constructs, with sequential microstates evaluated at consecutive GFP peaks maintaining stable spatial configurations while periodically exhibiting inverted polarities. This indicates that global microstates are formed by interacting neural populations [103]. This adaptive segmentation method captures quasi-stable states with average durations of 80 to 120 milliseconds. However, a limitation of this approach is that it exclusively focuses on GFP peak timepoints and excludes information generated at non-peak times [104].

To address the temporal limitations of GFP peak-focused approaches, subsequent studies were conducted using polarity-invariant modified k-means clustering to extract scalp topographies at GFP peak timepoints. The number of clusters (k) was determined to maximize the explanatory power for underlying data patterns [105,106]. For each time sample, the relabeling procedure for microstate classification assigns the class exhibiting the highest spatial correlation among predefined classes [107]. This involves a competitive back-fitting or winner-takes-all strategy [108], assigning a unique class label at every timepoint. This classification extends beyond GFP peak timepoints to all temporal points, ensuring temporal continuity. Timepoints that exhibit spatial correlation values below a threshold (r < 0.50) are marked as unassigned to preserve classification reliability [109]. This comprehensive approach allows for the analysis of spatial pattern dynamics over the entire recording duration, reflecting large-scale neural network coordination and cooperation patterns [10].

#### 3.4.2. Interpretation Methodology

Use of selective temporal sampling to address the temporal limitations of the GFP peak-focused approach may result in substantial loss of microstate information. Specifically, microstate templates extracted only at GFP peaks fail to capture neural activity transitions between peaks, potentially obscuring the actual temporal organization of neural state dynamics and limiting detection sensitivity for rapid state transitions [110].

Researchers have developed clustering methodologies for microstate extraction. A widely adopted approach involves the utilization of a modified version of the classical k-means algorithm that uses random starting configurations and multiple iterations to obtain optimal cluster designation. This stochastic clustering method performs iterative optimization to minimize the variance within clusters until convergence [105,107]. A deterministic hierarchical technique known as atomize and agglomerate hierarchical clustering (AAHC) initiates with a large number of clusters and progressively eliminates the cluster with the lowest global explained variance (GEV) by reassigning its constituents to the remaining clusters [106]. Although the modified k-means approach is computationally faster, it may yield slightly different topographies across runs due to its stochastic nature, whereas AAHC produces consistent results. Ideally, reported microstate alterations in tinnitus should be robust across these algorithmic differences. Notably, k-means clustering was employed in most of the reviewed studies and recent methodological comparisons indicate that microstate sequences derived from different clustering algorithms (e.g., k-means vs. AAHC) are information-theoretically invariant and yield highly consistent topographies [104,108]. Therefore, the consistent observation of altered microstates A and D coverage suggests that the core tinnitus-related microstate dynamics are robust neurophysiological features rather than artifacts of a specific clustering algorithm. Beyond these primary methods, investigators have implemented k-medoids clustering, in which each microstate representation corresponds to an actual topographical distribution from an EEG rather than derived or averaged topographies. Analyses of these various approaches have demonstrated that algorithm-specific factors partially influence static characteristics (such as GEV and inter-map spatial resemblance), whereas information-theoretic metrics and temporal dynamics (such as entropy rate, transition matrix convergence time, and sequential dependencies) exhibit considerable stability irrespective of the clustering method used [108].

Once a stable set of microstate topographies has been identified through clustering, the next step involves assigning these representative maps to the continuous EEG time series to capture their temporal evolution. The previously described backfitting procedure (also termed relabeling) is applied to each time sample, extending the classification from GFP peak timepoints to all temporal points to ensure temporal continuity in microstate assignment. Timepoints with spatial correlation values below a stringent threshold (r < 0.50) are considered unassigned to preserve classification reliability and minimize potential misclassification [107,109]. By maintaining these rigorous spatial correlation thresholds, this procedure ensures robust classification and enhances the validity of downstream temporal dynamics analyses. In addition, this comprehensive approach analyzes spatial pattern dynamics over the entire recording duration, reflecting large-scale neural network coordination and cooperation patterns [10]. As a result, microstate segmentation generally yields four canonical microstate classes (A, B, C, D) that collectively explain approximately 70–80% of the data variance, with most studies reporting approximately 79% [105,111,112,113]. These canonical classes reflect large-scale neural network coordination and cooperation patterns and demonstrate consistency across diverse populations and recording conditions, suggesting that they represent fundamental organizational principles of brain function.

These four canonical microstate classes correspond to specific functional neural networks with well-characterized anatomical substrates. Microstate A is predominantly observed in the left middle and superior temporal lobe, including Brodmann areas 41 (BA41, primary auditory cortex; Heschl’s gyrus) and 22 (BA22, Wernicke area), and the left insular cortex, with circumscribed but lower activation of the left lingual gyrus (BA19), collectively associated with the phonological processing network. Microstate B exhibits very strong activity in the bilateral occipital cortices (cuneus), including Brodmann areas 17 and 18 (primary visual cortex), with secondary smaller areas of activity in the right insular cortex extending to the right claustrum and right frontal eye field (BA8), consistent with the visual processing network. Microstate C shows major activity in the precuneus and the PCC, with weaker activation in the left angular gyrus, associated with the DMN. Microstate D demonstrates strong activation in the right inferior parietal lobe (BA40), right middle and superior frontal gyri, and right insula (BA13), reflecting the attention reorientation network and dorsal frontoparietal system [114,115].

GEV (%), mean duration (ms), coverage (%), frequency, and transition probabilities are calculated to quantify the spatiotemporal dynamics of EEG signals and interpret the functional and physiological significance of each microstate class [116]. GEV represents the percentage of variance in scalp potential maps explained by each microstate class. An increase in GEV indicates enhanced functional dominance of the associated neural network during the resting state, whereas a decrease reflects reduced network importance in aging populations or pathological conditions [117]. Mean duration, defined as the average time during which a microstate persists without interruption, reflects the stability and persistence of the underlying neural network state. Durations are longer during eyes-closed rest and NREM sleep, indicating slower and more stable network transitions [118]. Coverage, defined as the proportion of the total recording time occupied by each microstate class, increases with higher occurrence frequency of the microstate. For example, microstate A increases following antidepressant treatment, suggesting improved treatment response and normalized neural dynamics [119]. Frequency, which is the number of times a microstate appears per second, represents temporal dynamics in brain state transitions. Frequency increases during the hyperexcitation phase induced by propofol anesthesia and decreases in reduced consciousness or deep anesthesia states, reflecting altered network switching patterns [120,121]. Transition probabilities quantify the likelihood of switching from one microstate to another. Musically trained individuals display higher transition probability from microstate A to microstate B, indicating enhanced language–visual network interactions and integration of semantic processing. In contrast, non-trained individuals display higher transition probability from microstate A to microstate C and from microstate C to microstate D, reflecting increased recruitment of salient stimulus networks. This suggests that musical training reduces automatic attentional allocation to external stimuli and promotes more efficient cognitive state transitions [122].

EEG microstate analysis is broadly applied across diverse clinical and cognitive contexts. During a mental arithmetic task, microstate analysis successfully distinguishes high- and low-performing groups by comparing occurrence rates, mean GFP, and coverage of specific microstate classes [123]. In patients with Parkinson’s disease, changes in GEV before and after deep brain stimulation are significantly correlated with changes in MDS-UPDRS III scores, thereby validating microstate dynamics as a treatment-response biomarker [124]. In patients with subacute stroke, intermittent theta-burst stimulation to the lateral cerebellum increases the mean duration and coverage of microstate C and decreases these parameters for microstate D. These changes are significantly correlated with improvements in emotional and cognitive clinical scales, indicating applicability for intervention assessment and outcome prediction [125]. In AD, a decision-tree classifier based on increased mean durations of microstates C and D and decreased occurrence frequency of microstate B achieved 72% accuracy in disease classification, demonstrating the potential of microstate features as objective diagnostic biomarkers [126].

#### 3.4.3. Tinnitus-Specific Research Evidence

This section describes the synthesis of findings from four studies (N = 102 participants) on EEG microstates alterations in tinnitus populations (Table 3).

In patients with tinnitus, EEG microstates exhibit abnormal distributions and altered transition probabilities across multiple cortical networks, reflecting functional disruptions in the auditory and language processing, visual processing, and attentional control networks spanning the frontal, temporal, and parietal regions [15,127,128]. Microstate metrics obtained under eyes-open, eyes-closed, and mixed (eyes open/closed) conditions appear to show distinct characteristics and specific correlations with tinnitus symptoms [116]. In the eyes-closed condition, patients show markedly increased occurrence of microstate A compared to healthy controls, a finding that shows a positive correlation with tinnitus severity. In contrast, the mean duration, coverage, and frequency of microstate B increase under the mixed visual condition, exhibiting a negative correlation with tinnitus severity. Furthermore, the correlations between specific EEG microstate parameters and subjective tinnitus characteristics do not remain consistent across all visual conditions. In addition, statistically stronger and more significant associations have been observed in the eyes-closed and mixed conditions than in the eyes-open condition. Therefore, combining EEG microstate assessments conducted in the eyes-open, eyes-closed, and mixed conditions may potentially provide more sensitive neurophysiological markers for tinnitus evaluation.

EEG microstate characteristics observed in tinnitus patient populations appear to be modulated by clinical etiology. Patients with sudden sensorineural hearing loss accompanied by tinnitus exhibit reduced coverage, mean duration, and frequency of microstate A, and increased frequency of microstate B [15]. In contrast, patients with chronic subjective idiopathic tinnitus of unclear etiology demonstrate increases coverage of microstate A [116]. These findings suggest that the dynamic characteristics of microstate A in tinnitus patient populations may increase or decrease depending on the clinical etiology of tinnitus.

EEG microstate responses related to tinnitus-associated functional impairment may vary depending on clinical stage and symptom severity. A previous study indicated that in patients with sudden sensorineural hearing loss and tinnitus, THI score is negatively correlated with the frequency of microstate A and the transition probability from microstate D to A, and positively correlated with the transition probability from D to B [15]. In patients with chronic subjective idiopathic tinnitus, THI score is negatively correlated with the mean coverage, mean duration, and frequency of microstate D, and with the transition probability from B to D [116]. These observational findings suggest that functional impairment related to tinnitus may vary depending on clinical etiology and symptom characteristics.

Neurophysiological changes in EEG microstates following tinnitus intervention suggest the potential of objective indicators for tinnitus relief. Tailor-made notched music training (TMNMT) induces significant changes in EEG microstate dynamics under residual inhibition conditions, specifically inducing a reduction in the mean duration of microstate B, a decrease in the mean duration of microstate C, an increase in the frequency of microstates A and D, and an increase in the coverage of microstate A, and a significant increase in transition probabilities from A to B, from A to D, and from D to A [128]. These observed neurophysiological changes in EEG microstates suggest enhanced dynamic interactions between the auditory and attentional networks. Notably, a statistically significant positive correlation has been observed between tinnitus intensity and the mean duration of microstate B. This indicates that microstate B may serve as a potential neurophysiological indicator of tinnitus severity. If this relationship is proven to remain consistent across diverse visual conditions and independent samples, the mean duration of microstate B could be established as an objective and generalizable neurophysiological measure of tinnitus severity. Moreover, these findings imply that EEG microstate analysis may enhance clinical applicability, thereby contributing to comprehensive tinnitus assessment and treatment monitoring.

### 3.5. EEG Entropy

#### 3.5.1. Definition and Theoretical Foundation

Entropy is a concept that quantifies the degree of disorder or randomness in a system. It was originally formulated in thermodynamics to describe the state of gaseous or fluid systems based on the probabilistic distribution of molecular configurations. Highly ordered structures, such as crystals in which each molecule occupies a predetermined position, exhibit lower entropy than disordered systems, such as fluids with freely moving molecules. Claude E. Shannon adapted this concept to information theory, formalizing entropy as a quantitative measure of the irregularity, complexity, or unpredictability of signals [129]. Given that neuronal systems exhibit intrinsically nonlinear and, at times, chaotic dynamics, it is methodologically appropriate to apply tools based on nonlinear dynamics to EEG analysis [130]. Within this framework, entropy-based metrics are used to characterize the complex, nonlinear structure of EEG signals, thereby providing quantitative insights into the underlying physiological brain processes that cannot be fully resolved using conventional linear techniques.

Entropy measures for EEG analysis are broadly categorized into two families, one based on Shannon Entropy (ShEn) and the other rooted in Conditional Entropy (CoEn) [131]. The ShEn family quantifies the overall uncertainty or randomness in the distribution of a signal by measuring the average information obtained when observing a random variable. A prominent example is permutation entropy, which captures signal complexity by analyzing the ordinal patterns of data points [132]. In contrast, the CoEn family evaluates the uncertainty of one random variable based on the knowledge of another. This category includes widely used methods such as Approximate Entropy (ApEn) and Sample Entropy (SampEn), which evaluate signal regularity by assessing the self-similarity of repeated patterns [133]. Collectively, these complementary approaches provide a multifaceted picture of the nonlinear dynamics of the brain.

#### 3.5.2. Interpretation Methodology

ShEn (*H*) is a mathematical measure of the “choice” or “uncertainty” associated with the outcome of a process [129]. This measure is derived from three fundamental properties that any logical measure of uncertainty must satisfy. First, *H* must be a continuous function of the probabilities ($p_{i}$) for each outcome. Second, for equally likely outcomes, *H* must be a monotonic increasing function of the number of possible events (*n*), reflecting that more possibilities lead to greater uncertainty. Third, if a choice can be decomposed into two successive choices, the total *H* must be the weighted sum of the individual *H* values. The only function that satisfies all three of these properties is mathematically expressed using the following formula:

$$H\;=\;-\sum_{i=1}^{n}p_{i}\log_{2}p_{i}$$

where $p_{i}$ represents the probability of the *i*-th outcome. For the analysis of EEG data, the probability distribution ($p_{i}$) is derived from the signal’s PSD. Specifically, the power for each frequency band *i* (denoted as ${PW}_{i}$) is computed, and each probability $p_{i}$ is then determined by normalizing ${PW}_{i}$ against the total power of the entire spectrum [134]. This calculation yields the ShEn (*H*) value, which indicates the signal’s complexity. The interpretation of the resulting entropy value provides direct insight into the underlying neural dynamics. High *H* values indicate greater signal complexity and randomness, which are characteristic of active, information-rich cognitive states wherein signal power is broadly distributed across the frequency spectrum, making the neural activity less predictable. Conversely, low *H* values signify more ordered, structured, and predictable neural dynamics [135]. A study conducted to measure the EEG of patients with stroke indicated that the participants’ ShEn values, particularly in the delta band, tended to increase with increasing stroke severity [136]. This suggests that impairment of brain function increases with increasing stroke severity, leading to increased uncertainty and irregularity in EEG signals.

ApEn and SampEn are the most widely used entropy methods for assessing the complexity of biological data [137]. Both methods rely on three parameters: the embedding dimension (*m*), the tolerance criterion (*r*), and the time series length (*N*). The parameter *m* refers to the number of consecutive data points grouped as a pattern for comparison. The parameter *r* is the tolerance threshold for these comparisons. Patterns are considered to match when the distance between their corresponding points is less than *r*. *N* is the total length of the dataset used for analysis. ApEn is approximately the negative natural logarithm of the conditional probability (CP) that a dataset of length *N*, having repeated itself within a tolerance *r* for *m* points, will also repeat itself for *m* + 1 points [138]. This is mathematically expressed using the following formula:

$$ApEn\left(m,\;r,\;N\right)=\;\Phi^{m}\left(r\right)-\;\Phi^{m+1}(r)$$

The function *Φ^m^*(*r*) quantifies the prevalence of repetitive patterns within the time series. This is achieved by first constructing *N* − *m* *+* 1 overlapping vectors, each consisting of *m* consecutive data points. For every vector, a similarity probability, denoted as *C_i_^m^*(*r*), is calculated by determining the fraction of other vectors that fall within a specified tolerance *r*. The value of *Φ^m^*(*r*) is then defined as the average of the natural logarithms of these *C_i_^m^*(*r*) probabilities. This procedure is subsequently repeated for an increased dimension of *m* + 1 to compute *Φ^m^*^+^^1^(*r*). The final ApEn value is obtained by subtracting *Φ^m^^+^*^1^(*r*) from *Φ^m^*(*r*) [139].

This approach shows good reproducibility when applied to time series data, is robust to noise, and can detect changes in underlying episodic behavior that are not captured by peak occurrences or amplitudes [140]. Higher ApEn values indicate more irregular and less predictable signals, whereas lower values correspond to more periodic and stable signals. In a study that involved comparison of resting-state EEG between younger and older adults, the older adults exhibited higher ApEn in signals from the central, parietal, and occipital regions than their younger counterparts [141]. This indicates that physiological aging is associated with increased complexity and irregularity in brain dynamics, which may reflect a decline in the synchronization and connectivity of neural networks. However, ApEn inherently includes bias by counting each sequence as matching itself in its calculations. Additionally, ApEn is heavily dependent on record length and lacks relative consistency across different datasets [142].

SampEn was developed to reduce the inherent bias associated with ApEn. SampEn (*m*, *r*, *N*) is the negative natural logarithm of the CP that two sequences similar for *m* points remain similar at the next point, where self-matches are not included in calculating the probability [143]. The calculation of SampEn differs from ApEn in two major ways: it does not count self-matches and does not use a template-wise approach. Instead, SampEn calculates a single probability for the time series as a whole by first counting the total number of matching pairs of length *m*, and then the total number of matching pairs of length *m* + 1. This is mathematically expressed using the following formula:

$$SampEn\left(r,\;m,\;N\right)=\;-ln\frac{A^{m\;}(r)}{B^{m\;}(r)}$$

In this formula, *B^m^*(*r*) represents the probability that two sequences will match for *m* points, whereas *A^m^*(*r*) is the probability that they will match for *m +* 1 points. The ratio of these probabilities, *A^m^*(*r*)/*B^m^*(*r*), represents the CP that two sequences that match for *m* points will also match for *m* + 1 points in the entire dataset. Thus, SampEn assigns a non-negative value to the time series, with larger values indicating greater complexity and irregularity, and lower values denoting more self-similarity and predictability within the data. SampEn was used to quantify signal complexity in a study on the detection of real driving fatigue using EEG [144]. The results showed a decreased SampEn value for almost all channels in the fatigue state compared to the normal state. As this finding indicates that brain activity becomes more regular and less complex with the onset of driver fatigue, it supports the conclusion that SampEn is an “effectively distinguishing feature” for classifying normal and fatigue EEG signals.

#### 3.5.3. Tinnitus-Specific Research Evidence

This section describes the synthesis of findings from three studies (N = 36 participants) on EEG entropy alterations in tinnitus populations (Table 4).

Entropy measures such as ShEn, SampEn, and ApEn have been applied in quantitative analyses of EEG signals in tinnitus research [14,145,146]. The application of these non-linear methods stems from the recognition that brain dynamics, captured using EEG, reflect complex and chaotic activity that may not be fully captured in traditional linear analyses [147]. Research has shown that individuals with tinnitus exhibit entropy changes across the delta, theta, alpha, beta, and gamma frequency bands in several brain regions, such as the auditory, frontal, central, and parietal regions. Therefore, monitoring changes in specific EEG entropy patterns holds potential as an objective indicator for evaluating and tracking tinnitus.

The entropy characteristics of patients with tinnitus differ from those of normal controls. Studies have consistently demonstrated that patients with tinnitus exhibit higher entropy values in the delta and alpha frequency bands across brain regions such as the auditory, frontal, and central lobes than normal controls [14,145]. These common entropy patterns can be considered a reliable indicator of tinnitus-related changes in brain complexity. Research conducted using ShEn has revealed that patients with tinnitus consistently show elevated entropy across low and mid-frequency bands in the auditory, frontal, and central regions [14]. Similarly, analyses conducted using SampEn confirmed increased entropy in the delta, upper-alpha, and lower-beta bands across the auditory, frontal, central, and parietal lobes in tinnitus patient groups [145]. Furthermore, high ApEn values have been observed in the frontal and temporal lobes of patients with tinnitus, with a localized decrease at the C3 electrode site [146]. Elevated entropy values relative to control groups are interpreted as indicative of an increase in the relative complexity of the brain. However, this complexity should be interpreted with some nuance. This is because although increased entropy can signify an active, information-rich state in healthy neural processing, in the context of pathologies such as tinnitus, it more likely reflects “neural noise” or a dysregulated state of desynchronized firing [146]. This suggests that an entropy-based analysis of EEG frequency bands within specific brain regions, specifically observation of elevated complexity correlated with tinnitus distress, could serve as a potential biomarker of tinnitus, representing a shift toward pathological irregularity rather than functional complexity.

Existing tinnitus research has indicated that the efficacy of tinnitus relief could be evaluated by assessing objective changes in entropy patterns following therapeutic intervention. In a study that involved the administration of binaural beat sound therapy for one month, a statistically significant reduction in entropy was observed in low-frequency bands (delta, theta, and lower-alpha) in the tinnitus group. In addition, the post-treatment values of the treatment group approached those of a normal control group [14]. This post-treatment decrease in entropy suggests a reduction in neural complexity and a shift toward more regularized neural activity, indicating that ShEn can be used to measure treatment outcomes.

## 4. Discussion

EEG has the advantage of being able to serve as an objective indicator of tinnitus, which is typically assessed using subjective measurements. However, EEG only reflects cortical surface activity with relatively low spatial resolution; it cannot directly capture signals from deep brain structures such as the thalamus and amygdala, which are considered to play a central role in the pathophysiology of tinnitus [148]. Moreover, EEG has some critical technical limitations that must be considered. Volume conduction can spuriously inflate functional connectivity estimates, particularly in sensor–space analyses [86]. In addition, the choice of reference electrode (e.g., average vs. linked mastoid) can fundamentally alter power and coherence topographies [149]. Furthermore, EEG is highly sensitive to muscle and eye artifacts. Given that patients with tinnitus often present with high stress or anxiety levels, differential muscle tension artifacts between patient and control groups could potentially confound high-frequency (gamma/beta) findings [150,151]. Therefore, utilizing complementary neuroimaging techniques such as functional magnetic resonance imaging (fMRI) and positron emission tomography (PET) may be necessary for a comprehensive examination of structural and functional changes in tinnitus. Examinations conducted using PET have revealed increased synaptic density in the limbic system (amygdala, parahippocampal gyrus) and auditory regions (temporal lobe) of patients with chronic tinnitus, and decreased synaptic density in the frontal lobe, precuneus, and postcentral gyrus of patients with acute tinnitus. These findings suggest that hyperplasia occurs in some brain regions through the chronification of tinnitus from the initial stage of synaptic loss. Observation of this finding could confirm the presence of structural changes in the brains of patients with tinnitus [152]. Integration of these neuroimaging techniques and EEG to complement the low spatial resolution of EEG and the low temporal resolution of neuroimaging techniques may facilitate a more comprehensive understanding of the pathophysiology of tinnitus.

Various therapeutic interventions for tinnitus have been implemented. These include cognitive behavioral therapy (CBT), acceptance and commitment therapy, tinnitus retraining therapy, TMNMT, music therapy (MT), sound therapy, hearing aids, cochlear implants, and rTMS [153]. Notably, some important factors should be considered when evaluating these different therapeutic modalities using EEG. Cognitive fatigue can occur differentially depending on the type of task performed in therapy, resulting in changes in EEG rhythms, which make it difficult to quantify increases and decreases in specific frequency bands as integrated indicators of improvement in tinnitus symptoms [154]. In a study in which subjective questionnaire scores were compared with resting-state EEG data in CBT, MT, and CBT + MT groups, the subjective questionnaire scores of the CBT + MT group and the CBT group indicated significant alleviation of tinnitus. However, an EEG analysis revealed that the CBT + MT group showed changes in alpha and theta band power, whereas the CBT group showed increased gamma band power [155]. These findings suggest that to understand the treatment-specific EEG changes that could be indicators of tinnitus alleviation, a complementary approach that involves the analysis of correlations between EEG rhythm changes and tinnitus-related questionnaire scores should be considered for each therapeutic approach. In addition, EEG changes should be examined based on the underlying mechanisms of each therapeutic modality.

A major methodological concern in EEG-based tinnitus research is the interpretive limitations of single-metric analyses. Some previous studies have demonstrated that EEG signals can be affected by factors such as volume conduction or electromyographic contamination, indicating that single-metric interpretations have inherent limitations [77]. A study on moderation of tinnitus-related distress indicated that although feedback training induced clinical improvement of tinnitus perception in the study cohort, no significant EEG changes were observed in the PCC region [97]. Notably, the importance of combining multiple EEG indicators with clinical scales has been emphasized [156]. Collectively, these findings highlight the need to enhance the accuracy and reliability of analyses by adopting a multi-metric approach that incorporates power, connectivity, microstate, and entropy measures, rather than relying on a single EEG indicator. Moreover, concurrent use of validated clinical scales such as the THI or VAS may provide an essential basis for ensuring comparability and reproducibility across future studies.

Beyond technical limitations, interpretive uncertainty in EEG-based tinnitus analysis arises from the incomplete understanding of tinnitus-related networks and individual variability in neural adaptation. Furthermore, although effective connectivity analysis is useful for exploring causal relationships, excessive causal inference should be avoided when interpreting results, as the structure of tinnitus-related networks remains poorly defined [77,97]. This interpretive uncertainty may also be due to differences in neural adaptation patterns between patients. In previous studies, some patients showed clinical improvement without distinct EEG changes, whereas others demonstrated only limited symptom improvement despite exhibiting neurological alterations [97,156]. This suggests the need for personalized modeling in neurofeedback approaches. Moreover, such individual differences may require distinct modulation directions depending on whether the network is in a hyperconnected or hypoconnected state [95]. Specifically, some patients may require desensitization due to network hyperconnectivity, whereas others with weakened connectivity could require resensitization. These contrasting neural response patterns may partially account for the discrepancies observed across tinnitus studies. Therefore, implementation of personalized strategies tailored to specific frequency bands and network directions should be considered in future studies.

This review has several limitations that should be acknowledged. First, only a small number of the reviewed studies focused on emerging domains (e.g., only three and four studies were focused on entropy and microstates, respectively). This limits the generalizability of the findings specific to these emerging domains. Second, the possibility of publication bias cannot be completely ruled out. This is because studies in which “no difference” in EEG markers was observed between tinnitus and control groups are less likely to be published, potentially skewing the existing literature toward positive findings [157]. Third, methodological heterogeneity represents a significant challenge in synthesizing the current evidence. The electrode densities reported in the reviewed studies varied widely, ranging from standard 19-channel systems to high-density 128-channel arrays [158]. This disparity directly impacts the validity of source localization results, particularly for deep structures such as the limbic system, which is often implicated in tinnitus. Furthermore, variability in preprocessing rigor, such as the use of automated artifact rejection versus visual inspection, and the lack of consistent correction for multiple comparisons in connectivity analyses, may have affected the reliability of the reported between-group differences.

It is also important to consider the evolution of EEG methodology over the reviewed period (2000–2024). Earlier studies often relied on basic filtering and lower electrode densities, whereas recent investigations were conducted using advanced artifact rejection (e.g., ICA) and high-density source localization [158,159]. Despite these technical disparities, core findings such as gamma band enhancement and alpha reduction remained relatively consistent across the timeline. However, the more recent studies tended to indicate more spatially specific effects (e.g., differentiating parahippocampal vs. auditory cortex connectivity), likely reflecting improved spatial resolution and preprocessing rigor [160,161]. Future meta-analyses should account for “year of publication” or “preprocessing quality” as a moderator to quantify how methodological advances may influence effect sizes.

The evidence reviewed indicates that EEG-derived neurophysiological markers demonstrate associations with tinnitus presence and distress in group-level analyses. However, some critical gaps need to be addressed before these markers can be considered clinically validated biomarkers. No EEG metric has demonstrated sufficient sensitivity and specificity for individual-level diagnosis of tinnitus or prediction of treatment response, or any robust correlation with longitudinal symptom changes. Rigorous validation studies with adequate sample sizes, cross-validation using independent cohorts, and demonstration of test–retest reliability are necessary for the translation of statistically significant between-group differences into clinically useful diagnostic or prognostic tools. Notably, preliminary machine learning studies have achieved high classification accuracies (>90%) in distinguishing patients with tinnitus from controls [162]; however, studies with larger, more diverse samples and real-world clinical settings are required to replicate and confirm these findings. Therefore, although EEG markers show potential as research tools for understanding tinnitus pathophysiology, their clinical utility for diagnosis, treatment selection, or outcome monitoring remains unestablished.

Although the EEG domains discussed in this review were addressed separately, they likely reflect different facets of the same underlying pathophysiology [13]. The “Thalamocortical Dysrhythmia” model connects the observed increase in low-frequency power (delta/theta) to a compensatory increase in high-frequency activity (gamma) and “edge effect” synchronization [163]. This spectral shift likely manifests as the altered functional connectivity observed in long-range networks [164]. Furthermore, the disruption of stable auditory predictive coding may lead to the fragmentation of microstate dynamics (increased microstate A) and a consequent increase in signal entropy (system disorder) [127]. Future research should model these metrics simultaneously to validate this unified view of tinnitus as a disorder of large-scale neural network integration.

## 5. Conclusions

This narrative review synthesizes evidence from EEG-based studies of tinnitus across four analytical domains (spectral power, connectivity, microstates, and entropy) and shows that tinnitus is associated with distributed alterations in cortical oscillations, large-scale network interactions, and nonlinear signal complexity that extend beyond the auditory cortex. These group-level differences support the value of EEG as a research tool for investigating tinnitus pathophysiology and monitoring treatment-related brain changes. However, no EEG metric has demonstrated sufficient sensitivity, specificity, and reliability to serve as a standalone clinical biomarker for individual-level diagnosis, prognosis, or treatment selection in individuals with tinnitus. Rigorous, standardized, and multimodal studies are required to determine whether specific EEG-derived measures can achieve robust clinical utility in the future.

## Figures and Tables

**Table 2:** Summary of EEG connectivity analysis studies of tinnitus populations (the reviewed studies included those that reported quantitative connectivity analyses of patient and control groups, published 2000–2024).

Author(s) & Year of Publication	EEG Recording Configuration	Experimental Condition	EEG Analysis Method	Study Population (Sample Size)	Frequency Band(s)/Target Regions	Main Findings	Key EEG Indicators Identified in Tinnitus Studies
Mohan et al., 2016 [95]	19 electrodes (10–20 systems)	EC, RS	Filtering (2.0–44.0 Hz), Lagged phase coherence	TI(M): 210 TI(F): 101 NC(M): 154 NC(F): 102	δ (2.0–3.5 Hz), θ (4.0–7.5 Hz), α_1_ (8.0–10.0 Hz), α_2_ (10.0–12 Hz), β_1_ (13.0–18.0 Hz), β_2_ (18.5–21.0 Hz), β_3_ (21.5–30.0 Hz) γ (30.5–44.0 Hz)/frontal, parietal, temporal, and occipital	TI connectivity patterns: ↑θ in Right temporal↔Bilateral parietal ***; ↑β, γ in Fronto-limbic *** Global connectivity (all regions): ↑β, γ ***/↓δ, θ, α_1_ ***	TI characteristics: Theta connectivity increased between the right temporal and parietal regions, whereas beta and gamma connectivity increased within fronto-limbic networks. Overall, beta and gamma connectivity were elevated, whereas delta, theta, and alpha_1_ were reduced, reflecting frequency-dependent reorganization toward a white-noise-like pattern.
Xiong et al., 2023 [96]	128 electrodes (10–20 system)	EO, RS	Filtering (0.5–100.0 Hz & 50 Hz notch filtering), sLORETA; Lagged phase coherence	TI(M): 33 TI(F): 24 NC(M): 18 NC(F): 9	δ (2.0–3.5 Hz), θ (4.0–7.5 Hz), α_1_ (8.0–10.0 Hz), α_2_ (10.0–12.0 Hz), β_1_ (13.0–18.0 Hz), β_2_ (18.5–21.0 Hz), β_3_ (21.5–30.0 Hz), γ (30.5–44.0 Hz)/temporal, auditory cortex, frontal, prefrontal, INS, parietal cingulate, posterior cingulate, parahippocampus	TI (moderate-to-severe) vs. controls: ↑lagged coherence: PHC (right)–PCC (left) in β_3_ ***, Auditory cortex (right)–INS (right) in γ ** TI (moderate-to-severe) vs. (slight-to-mild): ↑INS–Auditory cortex in γ *** Correlation analysis (TI): INS (right)–PHC (left) connection↔THI (r > 0.37) in γ, INS(right)–PCC(left) connection↔THI (r > 0.37) in γ, Network: INS hub linking AN–SN–DMN ***	TI characteristics: Enhanced β_3_ coherence between parahippocampus and PCC (DMN–limbic coupling) and increased γ-band connectivity between the auditory cortex and insula, indicating an INS-centered hub integrating auditory, salience, and default mode networks. TI handicap: In the tinnitus group, γ-band connectivity among the insula, parahippocampus, and PCC, especially the INS–PHC and INS–PCC pathways, was positively correlated with THI, suggesting maladaptive auditory–salience–limbic interactions underlying tinnitus distress.
Vanneste et al., 2018 [97]	19 electrodes (10–20 system)	EC, RS. NF	Filtering (2.0–44.0 Hz), sLORETA source imaging; Phase-amplitude nesting; Granger causality	TI: 23 NC: 22	δ (2.0–3.5 Hz), θ (4.0–7.5 Hz), α (8.0–12.0 Hz), β (12.5–30.0 Hz), γ (30.5–44.0 Hz)/frontal, prefrontal, parietal cingulate, posterior cingulate, parahippocampus	PCC neurofeedback: ↓TQ distress post-training ***; ↓α–β, α–γ nesting post-training **; ↓functional connectivity: PCC–dACC, PCC–scACC, scACC–PHC; ↑PCC–PHC(α); ↓effective connectivity: PCC→dACC, PCC→scACC, PHC→scACC;	TI relief: PCC neurofeedback reduces α–β/α–γ nesting and both functional and effective connectivity within the distress network (PCC–dACC–scACC–PHC), while restoring α coupling between PCC and PHC.
Vanneste et al., 2012 [98]	19 electrodes (10–20 system)	EC, RS	Filtering (2.0–44.0 Hz), sLORETA, Lagged linear connectivity	TI(M): 18 TI(F): 18 NC(M): 18 NC(F): 18	δ (2.0–3.5 Hz), θ (4.0–7.5 Hz), α_1_ (8.0–10.0 Hz), α_2_ (10.0–12.0 Hz), β_1_ (13.0–18.0 Hz), β_2_ (18.5–21.0 Hz), β_3_ (21.5–30.0 Hz), γ (30.5–44.0 Hz)/prefrontal, orbitofrontal, PCC	TI (M) < TI (F) ↑α_1_ functional connectivity: Parahippocampus↔sgACC *, sgACC↔LINS *, LINS↔OFC (left) *, OFC (left)↔LA2 *, parahippocampus (left)↔RA1 *	TI characteristics: Sex-dependent pattern TI(F): increased α_1_ functional connectivity
De Ridder et al., 2011 [99]	19 electrodes (10–20 systems)	EC, RS	Filtering (0.5–45 Hz), sLORETA source localization, lagged phase coherence	TI: 55 NC: 84	δ (2.0–3.5 Hz), θ (4.0–7.5 Hz), α (8.0–12.0 Hz), β (13.0–30.0 Hz), γ (30.5–44.0 Hz)/AUD, BA 41, 42, STG, OFC, INS, ACC	TI: IC5 (sgACC-centered limbic network) & IC6(orbitofrontal-insular limbic network)-Connectivity: ↑α lagged coherence in parahippocampus↔sgACC↔orbitofrontal cortex↔inferior frontal gyrus; ↑β lagged coherence in parahippocampus↔sgACC↔orbitofrontal cortex↔inferior frontal gyrus	TI characteristics: Increased α–β connectivity in the sgACC–OFC–Ins–PHG limbic network; individuals with high-distress tinnitus exhibited stronger β power in the OFC–Ins–PHG component. The distress network shares the same topology as the pain and PTSD systems, suggesting a transdiagnostic affective–salience mechanism.

Methodological considerations: Sample sizes, electrode densities, and statistical rigor vary across studies. Effect sizes were not consistently reported in the original publications, limiting quantitative synthesis. Readers should interpret findings in consideration of these methodological variations. A1, primary auditory cortex; A2, secondary auditory cortex; AUD, auditory cortex; dACC, dorsal anterior cingulate cortex; EC, eyes closed; EO, eyes open; F, female; INS, insula cortex; M, male; NF, neurofeedback; NC, normal control group; OFC, orbitofrontal cortex; PCC, posterior cingulate cortex; PHC, parahippocampal cortex; RS, resting state; sgACC, subgenual anterior cingulate cortex; TI, individuals with tinnitus; vs., versus; δ, delta band; θ, theta band; α, alpha band; β, beta band; γ, gamma band; &, and; ↑/↓ denotes a significant difference (higher/lower) between compared groups; ↔ denotes bidirectional connectivity. *: *p* < 0.05; **: *p* < 0.01; ***: *p* < 0.001.

**Table 3:** Summary of EEG microstates analysis studies of tinnitus population (the reviewed studies included those that reported quantitative microstate analyses of patient and control groups, published 2000–2024).

Author(s) & Year of Publication	EEG Recording Configuration	Experiment Conditions	EEG Analysis Method	Study Population (Sample Size)	Frequency Band(s)/Target Regions	Main Findings				Key EEG Indicators Identified in Tinnitus Studies
						Coverage (%)	Mean Duration (ms)	Frequency	Transition Probability	
Cai et al., 2019 [15]	128 electrodes	EO	Filtering (2–20 Hz), modified k-means clustering algorithm	TI(TII): 25 NC: 27	A, B, C, D	TI(TII): ↓A *	TI(TII): ↓A *	TI(TII): ↓A *, ↑B*, THI↔A(r = −0.41) *	TI(TII): ↓(C→A) *, ↑(C→B) *, THI↔(D→A)(r = −0.447) *, THI↔(D→B)(r = 0.425) *	TI(TII) characteristics: decreased auditory network activation (↓A) and increased visual network compensation (↑B) TI(TII) handicap: altered default mode network transitions (↓C→A, ↑C→B) and reduced executive attention to auditory processing (↓D→A, ↑D→B). Higher tinnitus severity is correlated with greater auditory suppression and visual network reliance.
Wang et al., 2024 [116]	16 channel (10–20 system)	EO, EC, EOECm	Filtering (2–20 Hz), modified k-means clustering algorithm	TI: 18 NC: 18	A, B, C, D	TI(EOECm): ↑B *, ↓C *, TF↔A(r = 0.51) *, TF↔D(−0.58) *, Tinnitus intensity↔A(r = 0.53) *, Tinnitus intensity↔B(r = −0.52) *, THI↔D (r = −0.62) *	TI(EC): Tinnitus intensity↔A(r = 0.53) * TI(EOECm): ↑B *, ↓C *, Tinnitus intensity↔A(r = 0.58) *, Tinnitus intensity↔B(r = −0.53) *, TF↔D(r = −0.5) *, THI↔D(r = −0.65) *, OE vs. EC tinnitus: ↓B *, ↑D **	TI(EO): ↑A ** TI(EC): ↑A *, ↓D↔Tinnitus intensity(r = −0.68) ** TI(EOECm): ↑B *, Tinnitus intensity↔B(r = −0.51) *, Tinnitus intensity↔D(r = −0.59) *, THI↔D(r = −0.58) *, THI↔(B→D)(r = −0.64) *	TI(EC): ↑(B→A) *, ↑(C→A) *, ↑(D→A) *, Tinnitus intensity↔(B→D)(r = −0.5) *, Tinnitus intensity↔(C→A)(r = −0.5) * EO: ↓(A→B) *, ↓(C→B), ↓(D→B) * TI(EOECm): ↑(B→A) *, ↑(C→A) *, ↑(D→A) *, ↓(C→D) *, ↓(D→C) *, Tinnitus intensity↔(A→C)(r = 0.6) *, Tinnitus intensity↔(C→A)(r = 0.59) *, Tinnitus intensity↔(D→B)(r = −0.65) *, TF↔(B→A)(r = 0.5) *, TF↔(C→A)(r = 0.51) *, TF↔(C→D)(r = −0.55) *, THI↔(B→D)(r = −0.64) *	TI handicap (EC): Increased activity in the auditory network (↑A) and more frequent transitions from other networks to auditory processing are linked to higher tinnitus intensity. This means the brain is more focused on hearing, which worsens tinnitus symptoms. TI relief (EOECm): Greater visual network engagement (↑B: longer duration, higher coverage, and more frequent occurrence) is correlated with decreased tinnitus intensity (r = −0.51). This indicates the brain’s attention shifts toward visual processing, helping to relieve tinnitus. TI characteristics (EOECm): Increased transitions from the default mode network (C) to the auditory network (A) is correlated with increased tinnitus intensity and frequency (↑C→A)(r = 0.59, r = 0.51). This reflects central neural reorganization whereby heightened reliance on auditory processing amplifies tinnitus perception.
Cai et al., 2018 [127]	128 electrodes	EO	Filtering (2–20 Hz), modified k-means clustering algorithm	TI: 15 NC: 17	A, B, D	TI: ↑A ***, ↓D *	TI: ↑A **, ↓D *, TL↔C(r = 0.57) *	-	TI: ↓(D→B) *	TI characteristics: central reorganization with auditory (↑A), executive (↓D), and default mode network (↑C) dysfunction, along with reduced (↓D→B) transitions indicating impaired attention shifting
Zhu & Gong, 2023 [128]	64 electrodes (10–20 system)	EC, TMNMT	AAHC	TI(TMNMT): 22 TI-Sham: 22	A, B, D	TI(TMNMT): ↑A **, ↓C *	TI(TMNMT): ↓B *, ↓C **, B↔TFI(r = 0.54) *	TI(TMNMT): ↑A **, ↑C *, ↑D *	TI(TMNMT): ↑(A→B) *, ↑(A→D) **, ↑(D→A) *, ↓(D→B) *	TI relief: increased auditory network (↑A) and attention network (↑D) activity, with reduced visual network (↓B), wherein B mean duration is positively correlated with TFI severity (r = 0.54)

Methodological considerations: Sample sizes, electrode densities, and statistical rigor vary across studies. Effect sizes were not consistently reported in the original publications, limiting quantitative synthesis. Readers should interpret findings in consideration of these methodological variations. A, microstates class A; AAHC, atomize and agglomerate hierarchical clustering; B, microstates class B; C, microstates class C; D, microstates class D; EC, eyes closed; EO, eyes open; EOECm, eyes-opening, eyes-closing mixed; NC, normal control group; TF, tinnitus frequency; TFI, tinnitus functional index; THI, tinnitus handicap inventory; TI, individuals with tinnitus; TII, individuals with tinnitus and ISSNHL; TL, tinnitus loudness; TMNMT, tailor-made notched music training; vs.: versus; ↑/↓ denotes a significant difference (higher/lower) between compared groups; → denotes transition probability between states; ↔ denotes bidirectional correlation. *: *p* < 0.05; **: *p* < 0.01; ***: *p* < 0.001.

**Table 4:** Summary of EEG entropy analysis studies of tinnitus populations (the reviewed studies included those that reported quantitative entropy analyses of patient and control groups, published 2000–2024).

Author(s) & Year of Publication	EEG Recording Configuration	Experiment Conditions	EEG Analysis Method	Study Population (Sample Size)	Frequency Band(s) /Target Regions	Main Findings	Key EEG Indicators in Tinnitus Studies
Sadeghijam et al., 2021 [14]	32 electrodes (10–20 system)	EC, BBT	Filtering (0.4–256 Hz), QEEG, ShEn	TI: 19 NC: 23	δ, θ, lower-α, upper-α, lower-β, mid-β, upper-β, γ/right auditory, left auditory, right frontal, left frontal, central	Pre-Intervention (TI): ↑δ, θ, lower-α, upper-α across all ROIs *; ↑lower-β in right auditory, right frontal, central only * Post-Intervention (TI): ↓δ in right auditory, right frontal, left frontal ROIs *; ↓θ, lower-α across all ROIs *; ↓upper-α in left frontal only * (despite these decreases, still higher than controls)	TI characteristics: Entropy-dominant pattern (↑δ, θ, lower-α, upper-α across ROIs; ↑low-β in right auditory, right frontal, central) TI relief: post-intervention partial normalization (↓δ in right auditory/right frontal/left frontal; ↓θ, lower-α globally; ↓upper-α in left frontal only); despite these reductions, the patients still exhibited higher values than the controls.
Jianbiao et al., 2022 [145]	64 electrodes	RS	Filtering (0.5–80 Hz), Notch Filter (49.5–50.5 Hz), SampEn	TI: 10 NC: 10	δ (0.5–3.5 Hz), θ (4–7.5 Hz), lower-α (8–10 Hz), upper-α (10–12 Hz), lower-β (13–18 Hz), mid-β (18.5–21 Hz), upper-β (21.5–30 Hz), γ (30.5–44 Hz)/right frontal, left frontal, right auditory, left auditory, central, right parietal lobe, left parietal lobe	SampEn TI: ↑δ in right parietal lobe **; ↑upper-α in left auditory *, central **, left parietal lobe *; ↑θ in right parietal lobe *; ↓θ in left parietal lobe *; ↓lower-α in central *; ↓γ in left auditory *, left frontal **	TI characteristics: Comparisons of mean values with the NC group showed that the TI exhibited higher mean entropy in (↑δ, upper-α, lower-β) rhythms across all analyzed ROIs. The TI showed lower mean entropy in (↓θ, lower-α, mid-β, upper-β, γ) rhythms in most ROIs.
Naghdabadi & Jahed, 2024 [146]	64 electrodes (10–20 system)	RS, EO	Filtering (0.4–200 Hz), ApEn	TI: 7; NC: 7	-/AG, dlPFC, MTG, OFC, S1, SMA, SMG, STG, vlPFC, cuneal cortex & precuneus	ApEn TI: ↑Entropy in OFC(FPz, FP2) ***; dlPFC(F3, F4) ***; SMA(Fz, FC3, FCz, FC4, FT8) ***; MTG & STG(T7, T8) ***; SMG(CP3) ***; AG(P3, P4, CP4) ***; cuneal cortex & precuneus(POz) ***; ↓Entropy in C3 *	TI characteristics: characterized by widespread increased complexity (↑ApEn in frontal, temporal) alongside a focal decrease (↓ApEn in C3).

Methodological considerations: Sample sizes, electrode densities, and statistical rigor vary across studies. Effect sizes were not consistently reported in the original publications, limiting quantitative synthesis. Readers should interpret findings in consideration of these methodological variations. AG, angular gyrus; ApEn, approximate entropy; BBT, binaural beat therapy; dlPFC, dorsolateral prefrontal cortex; EC, eyes closed; EO, eyes open; MTG, middle temporal gyrus; NC, normal control group; OFC, orbitofrontal cortex; QEEG, quantitative electroencephalographic; ROI, regions of interest; RS, resting state; S1, primary somatosensory cortex; SampEn, sample entropy; ShEn, Shannon entropy; SMA, supplementary motor area; SMG, supramarginal gyrus; STG, superior temporal gyrus; TI, individuals with tinnitus; vlPFC, ventrolateral prefrontal cortex; α, alpha band; β, beta band; γ, gamma band; δ, delta band; θ, theta band; ↑/↓ denotes a significant difference (higher/lower) between compared groups. *: *p* < 0.05; **: *p* < 0.01; ***: *p* < 0.001.

## Data Availability

No new data were created or analyzed in this study.

## Abbreviations

The following abbreviations are used in this manuscript:

EEG	Electroencephalography
THI	Tinnitus Handicap Inventory
VAS	Visual Analog Scales
PSD	Power Spectral Density
STFT	Short-Time Fourier Transform
QEEG	Quantitative Electroencephalography
AD	Alzheimer’s Dementia
TQ	Tinnitus Questionnaire
rTMS	Repetitive Transcranial Magnetic Stimulation
PLV	Phase-Locking Value
PCC	Posterior Cingulate Cortex
DMN	Default Mode Network
GFP	Global Field Power
AAHC	Atomize and Agglomerate Hierarchical Clustering
GEV	Global Explained Variance
NREM	Non-Rapid Eye Movement
TMNMT	Tailor-Made Notched Music Training
ShEn	Shannon Entropy
CoEn	Conditional Entropy
ApEn	Approximate Entropy
SampEn	Sample Entropy
TP	Total Power
CP	Conditional Probability
fMRI	Functional Magnetic Resonance Imaging
PET	Positron Emission Tomography
CBT	Cognitive Behavioral Therapy
MT	Music Therapy
